# The histone demethylase KDM5 controls developmental timing in *Drosophila* by promoting prothoracic gland endocycles

**DOI:** 10.1242/dev.182568

**Published:** 2019-12-20

**Authors:** Coralie Drelon, Michael F. Rogers, Helen M. Belalcazar, Julie Secombe

**Affiliations:** 1 Department of Genetics, Albert Einstein College of Medicine, 1300 Morris Park Avenue, Bronx, NY 10461, USA; 2 Dominick P. Purpura Department of Neuroscience, Albert Einstein College of Medicine, 1410 Pelham Parkway South, Bronx, NY 10461, USA

**Keywords:** KDM5, Lid, Transcription, Prothoracic gland, Endocycle, Torso, MAPK pathway, Ecdysone, *Drosophila*

## Abstract

In *Drosophila*, the larval prothoracic gland integrates nutritional status with developmental signals to regulate growth and maturation through the secretion of the steroid hormone ecdysone. While the nutritional signals and cellular pathways that regulate prothoracic gland function are relatively well studied, the transcriptional regulators that orchestrate the activity of this tissue remain less characterized. Here, we show that lysine demethylase 5 (KDM5) is essential for prothoracic gland function. Indeed, restoring *kdm5* expression only in the prothoracic gland in an otherwise *kdm5* null mutant animal is sufficient to rescue both the larval developmental delay and the pupal lethality caused by loss of KDM5. Our studies show that KDM5 functions by promoting the endoreplication of prothoracic gland cells, a process that increases ploidy and is rate limiting for the expression of ecdysone biosynthetic genes. Molecularly, we show that KDM5 activates the expression of the receptor tyrosine kinase *torso*, which then promotes polyploidization and growth through activation of the MAPK signaling pathway. Taken together, our studies provide key insights into the biological processes regulated by KDM5 and expand our understanding of the transcriptional regulators that coordinate animal development.

## Abstract (precis)

**Summary:** Identification of KDM5 as a new transcriptional regulator of the MAPK signaling cascade provides insights into the molecular mechanisms governing the regulation of ecdysone production and developmental growth control.

## INTRODUCTION

One key means by which transcription is regulated is through changes to covalent modifications that occur on nucleosomal histone proteins that comprise chromatin (Bannister and Kouzarides, 2011). The enzymes that add or remove these modifications play crucial roles during development and their dysregulation can lead to a number of different human disorders (Mirabella et al., 2016). Lysine demethylase 5 (KDM5) family proteins are chromatin-mediated regulators of transcription that are encoded by four paralogous genes in mammalian cells, KDM5A, KDM5B, KDM5C and KDM5D, and by a single gene in *Drosophila*, *kdm5* (also known as *little imaginal discs*; *lid*). The most well-established gene regulatory function of KDM5 proteins is their enzymatic activity, which demethylates trimethylated lysine 4 of histone H3 (H3K4me3) (Accari and Fisher, 2015; Xhabija and Kidder, 2018). High levels of H3K4me3 are found surrounding transcriptional start sites and are associated with active gene expression (Santos-Rosa et al., 2002). Whereas absolute levels of H3K4me3 are unlikely to be primary drivers of gene expression levels, the breadth of these promoter peaks can impact transcriptional consistency (Benayoun et al., 2014; Howe et al., 2017). Recruitment of KDM5 to promoters to demethylate H3K4me3 is therefore one mechanism by which this family of proteins regulates transcription. KDM5 proteins can also affect gene expression through demethylase-independent mechanisms, such as through interactions with the chromatin remodeling NuRD complex or by regulating histone acetylation via interactions with lysine deacetylase (HDAC) complexes (Barrett et al., 2007; Gajan et al., 2016; Lee et al., 2009; Liu et al., 2014; Nishibuchi et al., 2014).

KDM5 proteins play key roles in orchestrating diverse gene expression programs. This is emphasized by the large volume of literature linking dysregulation of KDM5 proteins to two seemingly disparate clinical outcomes: cancer and intellectual disability. Whole-exome sequencing of individuals with intellectual disability has identified loss of function mutations in KDM5A, KDM5B and KDM5C (Collins et al., 2019; Kim et al., 2017; Vallianatos and Iwase, 2015). Efforts to understand the link between KDM5 and intellectual disability using mice, flies and worms have revealed that KDM5 can regulate many genes that impact neuronal development or function (Chen et al., 2019; Iwase et al., 2016; Mariani et al., 2016; Zamurrad et al., 2018). However, the extent to which any of these pathways contribute to the cognitive impairments observed in patients remains unknown. A similar deficit exists in our understanding of how the dysregulation of human KDM5 genes contributes to cancer (Blair et al., 2011; Plch et al., 2019). In contrast to intellectual disability, which is exclusively associated with loss-of-function mutations in KDM5 genes, malignancies have been associated with overexpression of KDM5A or KDM5B, either loss or gain of KDM5C, or loss of KDM5D. The best studied of these is the overexpression of KDM5B observed in breast cancer and melanoma, which correlates with poor prognosis (Han et al., 2017). Despite being shown to directly or indirectly regulate genes involved in cell cycle progression, cancer stem cell survival and DNA repair, no clear model has emerged to explain its oncogenic capacities (Catchpole et al., 2011; Han et al., 2017; McCann et al., 2019; Roesch et al., 2013; Yamamoto et al., 2014; Yamane et al., 2007). Our lack of understanding of KDM5-regulated pathways that lead to clinical disorders underscores the importance of defining the physiological functions of KDM5 proteins.

*Drosophila melanogaster* offers a genetically amenable model for providing fundamental insight into KDM5 function *in vivo*, as it encodes a single, highly conserved, *kdm5* gene (Gildea et al., 2000). Moreover, in contrast to knockouts of KDM5A, KDM5B or KDM5C in mice that are homozygous viable, loss of *Drosophila kdm5* results in lethality (Albert et al., 2013; Drelon et al., 2018; Iwase et al., 2016; Klose et al., 2007; Martin et al., 2018). This allows us to dissect crucial functions of KDM5 without the complication of functional redundancy between mammalian KDM5 paralogs that could partially occlude phenotypes. We have previously shown that *kdm5* null mutants take 5 days longer than wild-type animals to complete larval development, linking KDM5 function to growth control. Interestingly, this phenotype is independent of the histone demethylase activity of KDM5, as animals specifically lacking this enzymatic function grow normally and produce viable adult flies (Drelon et al., 2018; Li et al., 2010). Emphasizing the importance of understanding cellular functions of KDM5 proteins that are independent of their enzymatic activity, the contribution of the demethylase function of KDM5 to normal development and to clinical disorders in mammalian cells remains unresolved. For example, although some intellectual disability-associated mutations in KDM5C reduce *in vitro* histone demethylase activity, others do not (Brookes et al., 2015; Iwase et al., 2007; Tahiliani et al., 2007; Vallianatos et al., 2018). Similarly, whereas the growth of some cancers can be attenuated by pharmacologically inhibiting KDM5 demethylase activity, KDM5 appears to act through demethylase-independent activities in others (Cao et al., 2014; Paroni et al., 2018). KDM5 proteins are therefore likely to use more than one gene-regulatory activity to control a range of cellular processes *in vivo.*

Here, we demonstrate that KDM5 regulates larval development by playing an essential role in the prothoracic gland, a tissue that secretes the steroid hormone ecdysone, which is a key regulator of animal growth and maturation (Yamanaka et al., 2013). Although KDM5 is normally expressed ubiquitously during larval development, re-expression of *kdm5* exclusively in the prothoracic gland within a *kdm5* null mutant background rescues the larval growth delay and restores adult viability. We further show that within cells of the prothoracic gland, KDM5 is necessary to promote the endoreplicative cell cycles that increase DNA copy number and is required for the transcription of enzymes that mediate ecdysone production (Ohhara et al., 2017). Crucial to the prothoracic gland functions of KDM5 is its regulation of the Torso receptor tyrosine kinase, which activates the MAPK pathway to trigger ecdysone production (Rewitz et al., 2009; Shimell et al., 2018). By identifying KDM5 as a new transcriptional regulator of this signaling cascade, our data provide new insights into the molecular mechanisms governing the regulation of ecdysone production and developmental growth control.

## RESULTS

### KDM5 expression in the prothoracic gland is sufficient to rescue lethality and restore correct developmental timing to *kdm5* null mutants

**Fig. 1.**
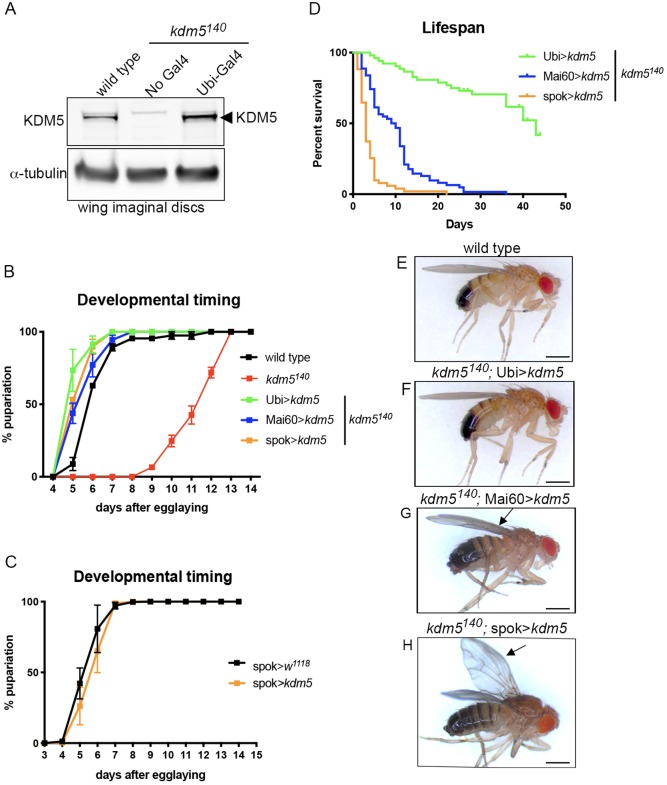
**KDM5 expression in the prothoracic gland rescues the *kdm5^140^* developmental delay and lethality.** (A) Western blot using 3rd instar larval wing imaginal discs (five per lane) from wild type, *kdm5^140^* (no Gal4 driver) and *kdm5^140^* expressing *kdm5* ubiquitously (*kdm5^140^*; Ubi>*kdm5*). Anti-KDM5 (top panel) is indicated by the arrowhead. Anti-α-tubulin is used as a loading control (bottom panel). (B) Number of days taken for pupariation to occur in wild type (*n=*82), *kdm5^140^* (*n=*49), *kdm5^140^*; Ubi>*kdm5* (*n=*69), *kdm5^140^*; Mai60>*kdm5* (*n=*63) and *kdm5^140^* ; spok>*kdm5* (*n=*74). Data are mean±s.e.m. (C) Number of days taken for pupariation to occur in control (spok>*w^1118^*; *n=*417) and spok>*kdm5* animals (*n=*666). Data are mean±s.e.m. (D) Adult survival for *kdm5^140^*; Ubi>*kdm5* (*n=*56), *kdm5^140^*; Mai60>*kdm5* (*n=*62) and *kdm5^140^*; spok>*kdm5* (*n=*51) rescued male flies. Mai60 and spok-Gal4-rescued flies are significantly shorter lived than Ubi-Gal4-rescued flies (Mantel-Cox log-rank test; *P*<0.0001). (E) Male wild-type fly (*kdm5^140^*; g[*kdm5*:HA]attp86F). (F) Male *kdm5^140^*; Ubi>*kdm5* adult fly showing morphologically normal features. (G) Male *kdm5^140^*; Mai60>*kdm5* adult fly with normal body and slightly curved wings indicated by an arrow. (H) Male *kdm5^140^*; spok>*kdm5* adult fly with normal body and curved wings indicated by an arrow. Scale bars: 500 µm.

To understand the underlying basis of the lethality caused by the *kdm5^140^* null allele, we sought to define the spatial requirements of KDM5 function during development. To do this, we re-expressed *kdm5* within different tissues of the null mutant using a Gal4-inducible transgene that we have previously used to rescue hypomorphic alleles of *kdm5* (Li et al., 2010) (Table 1). Ubiquitous expression of this UAS-*kdm5* transgene using Ubiquitin-Gal4 (Ubi-Gal4) resulted in slightly higher than endogenous levels of KDM5 expression and rescued the lethality of *kdm5^140^* (Fig. 1A; Table 1). Re*-*expressing *kdm5* ubiquitously also led to a developmental timing profile that was similar to that of wild-type flies (Fig. 1B). This is consistent with the observation by us and others that KDM5 is broadly expressed in all cell types examined to date (Lee et al., 2009; Liu et al., 2014; Moshkin et al., 2009; Secombe et al., 2007; Tarayrah et al., 2015; Zamurrad et al., 2018).

**Table 1.**
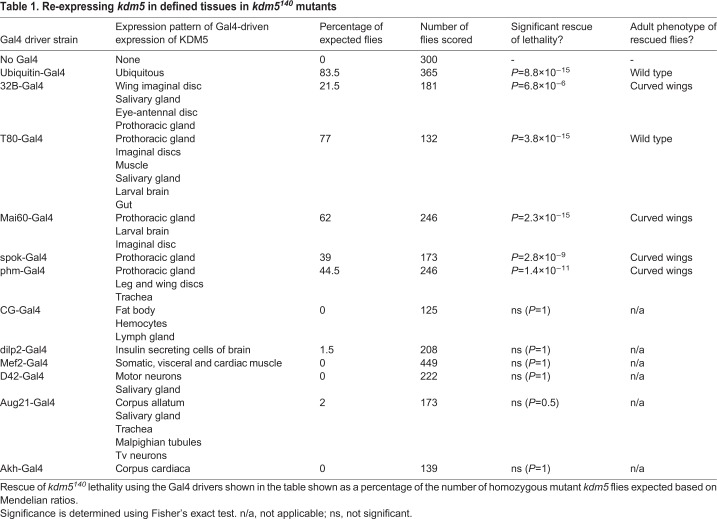
**Re-expressing *kdm5* in defined tissues in *kdm5^140^* mutants**

To test whether KDM5 plays key developmental roles in specific tissues, we tested a range of Gal4 drivers for their ability to rescue *kdm5^140^* lethality when combined with UAS-*kdm5* (Table 1). These included strains that drive Gal4 expression in tissues that have phenotypes in *kdm5* mutant or knockdown larvae, such as imaginal discs and hemocytes (Drelon et al., 2018; Morán et al., 2015; Moshkin et al., 2009). In addition, we tested tissues linked to the regulation of larval growth, such as the hormone-producing larval prothoracic gland, insulin secreting cells of the brain and cells of the fat body that coordinate larval growth with feeding and nutritional status (Yamanaka et al., 2013). Significantly, all Gal4 drivers that were expressed in the larval prothoracic gland significantly rescued *kdm5^140^* lethality, including spookier-Gal4 (spok-Gal4), which is expressed exclusively in this tissue (Table 1) (Hrdlicka et al., 2002; Moeller et al., 2017; Shimell et al., 2018). Consistent with KDM5 playing crucial functions in the ecdysone-secreting larval prothoracic gland, spok-Gal4-mediated re-expression of *kdm5* was sufficient to rescue the developmental delay *kdm5^140^* (Fig. 1B). Interestingly, UAS-mediated expression of *kdm5* in a wild-type background did not accelerate normal growth rate, suggesting that KDM5 is necessary but not sufficient for prothoracic gland function during larval development (Fig. 1C).

It is notable that while expression of *kdm5* in the prothoracic gland allowed *kdm5^140^* mutant animals to eclose, adult flies were shorter-lived than flies expressing *kdm5* ubiquitously (Fig. 1D). In addition, while adults rescued by ubiquitous expression of *kdm5* were largely male and female fertile, prothoracic gland re-expression of *kdm5* resulted in significant infertility (Table 2). These data are consistent with previous observations showing roles for KDM5 in oogenesis and in testis germline stem cell proliferation (Navarro-Costa et al., 2016; Tarayrah et al., 2015; Zhaunova et al., 2016). Together, these studies demonstrate essential roles for KDM5 in the larval prothoracic gland and in other cell types that are important for adult survival and reproduction.

**Table 2.**
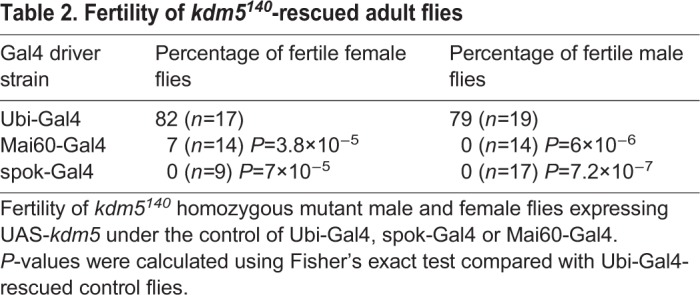
**Fertility of *kdm5^140^*-rescued adult flies**

**Fig. 2.**
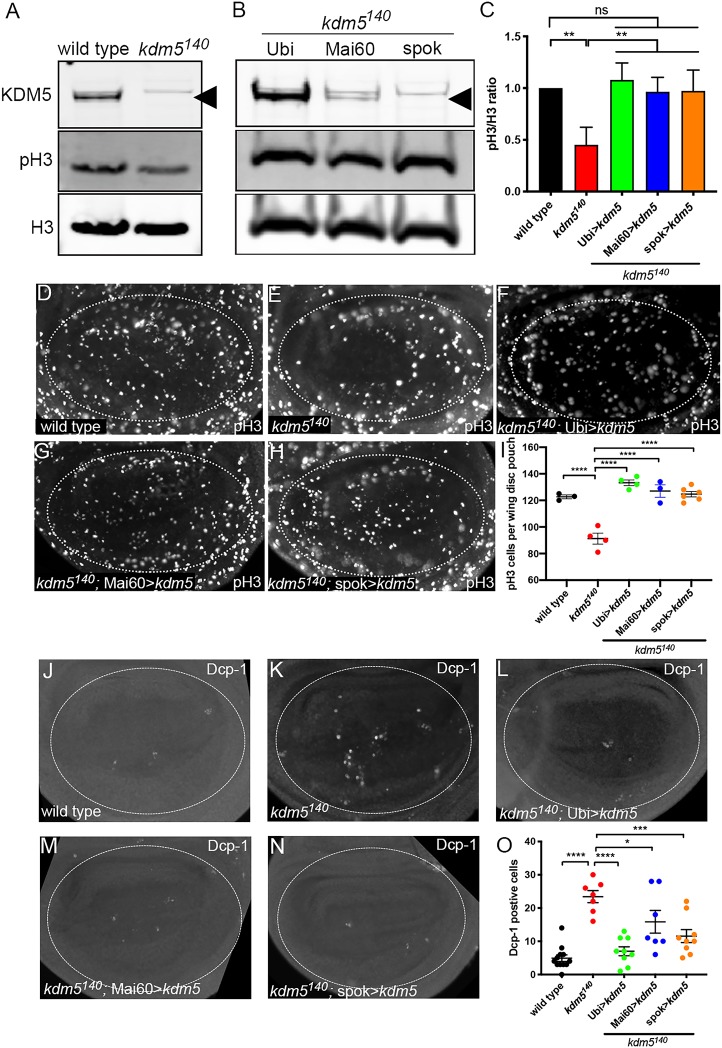
**Increased cell death and reduced proliferation in *kdm5^140^* wing discs is due to non-cell autonomous effects.** (A) Western blot using anti-KDM5 (top), anti-phosphorylated histone H3 (middle; pH3) and total histone H3 (bottom; H3) in wild-type and *kdm5^140^* wing discs. Six wing discs per lane. (B) Western blot using wing discs from *kdm5^140^* mutants re-expressing *kdm5* using Ubi-Gal4, Mai60-Gal4 or spok-Gal4. Anti-KDM5 (top panel), pH3 (middle panel) and total H3 (bottom panel). Six wing discs per lane. Arrowheads indicate KDM5. (C) Quantification of the levels of pH3 relative to total H3 from three western blots. **P<0.01 (one-way ANOVA); ns, not significant. Data are mean± s.e.m. (D-H) Anti-pH3 staining of wing imaginal disc from (D) a wild-type wandering 3rd instar larva, (E) a *kdm5^140^* wandering 3rd instar larva, (F) *kdm5^140^*; Ubi>*kdm5* wandering 3rd instar larva, (G) a *kdm5^140^*; Mai60>*kdm5* wandering 3rd instar larva and (H) a *kdm5^140^*; spok>*kdm5* wandering 3rd instar larva. Dotted oval indicates wing pouch region used for quantification. (I) Quantification of the number of pH3-positive cells in the pouch region of wild type (*n=*3), *kdm5^140^* (*n=*4), *kdm5^140^*; Ubi>*kdm5* (*n=*4), *kdm5^140^*; Mai60>*kdm5* (*n=*3), *kdm5^140^*; spok>*kdm5* (*n=*6). ****P<0.0001 (one-way ANOVA). Data are mean±s.e.m. with individual data points indicated. (J-N) A wild-type wing imaginal disc (J), a *kdm5^140^* mutant wing imaginal disc (K), a wing imaginal disc from *kdm5^140^*; Ubi>*kdm5* (L), a wing imaginal disc from *kdm5^140^*; Mai60>*kdm5* (M) and a wing imaginal disc from *kdm5^140^*; spok>*kdm5* (N) from wandering 3rd instar larvae stained using the apoptosis marker anti-Dcp-1. Dotted circle indicates pouch region of the wing disc that was used to count Dcp-1-positive cells. (O) Quantification of the number of Dcp-1 positive cells in the pouch region of wing imaginal discs from wild type (*n=*12), *kdm5^140^* (*n=*7), *kdm5^140^*; Ubi>*kdm5* (*n=*9)*, kdm5^140^*; Mai60>*kdm5* (*n=*7) and *kdm5^140^*; spok>*kdm5* (*n=*8)*.* ****P<0.0001, ***P<0.001, *P<0.05 (one-way ANOVA). Data are mean±s.e.m. with individual data points indicated.

These rescue studies also revealed a role for KDM5 in wing development. While adult flies generated by re-expression of *kdm5* in the prothoracic gland, such as spok-Gal4, were the same size as those rescued by ubiquitous expression, they had wings that were curved downward compared with wild type (Fig. 1E-H). A similar curved-down wing phenotype was observed in *kdm5^140^* flies that expressed *kdm5* in the wing imaginal disc in addition to the prothoracic gland such as Mai60-Gal4 or 32B-Gal4, but not with more broadly expressed drivers such as T80-Gal4 (Fig. 1G, Table 1). This curved-wing phenotype could be caused by the proliferative or cell death phenotypes we previously observed in *kdm5^140^* larval wing imaginal discs (Drelon et al., 2018). To test this, we quantified wing disc proliferation and cell death using antibodies that detect the mitotic marker phosphorylated histone H3 (pH3) and the cleaved caspase Dcp-1, respectively. As expected, the decreased levels of pH3 observed by western blot and whole-mount staining of *kdm5^140^* wing imaginal discs were fully restored by ubiquitous *kdm5* expression using Ubi-Gal4 (Fig. 2A-I). The reduced levels pH3 seen in *kdm5^140^* were similarly rescued by expressing *kdm5* under the control of Mai60-Gal4 or spok-Gal4 that drives low or no KDM5 expression in the wing disc, respectively (Fig. 2B-I). Likewise, the increased number of Dcp-1-positive cells seen in *kdm5^140^* wing discs was restored to wild-type levels by Mai60-Gal4 or spok-Gal4-driven expression of *kdm5* (Fig. 2J-O). Thus, neither the proliferative nor the apoptotic phenotypes of *kdm5^140^* wing imaginal discs are triggered by loss of KDM5 in the disc itself but are caused by cell-nonautonomous mechanisms. Although the basis for the wing defects observed in Mai-60 and spok-Gal4 rescued flies remains unclear, it could be due to a requirement for KDM5 during pupal development or during wing maturation in newly eclosed flies.

### Reduced ecdysone production causes the developmental delay of *kdm5^140^* mutant larvae

The prothoracic gland, corpus allatum and corpora cardiaca are the three sub-tissues that make up the larval ring gland: a semi-circular structure associated with the central larval brain. Whereas the prothoracic gland synthesizes ecdysone, the corpus allatum and corpora cardiaca produce juvenile hormone and adipokinetic hormone, respectively (Sánchez-Higueras et al., 2014). In order to evaluate the role of KDM5 in these two other sub-tissues, we tested whether expressing *kdm5* in the corpus allatum rescued the lethality of *kdm5^140^* using Aug21-Gal4 (Table 1) (Adam et al., 2003; Siegmund and Korge, 2001). In contrast to expression in the prothoracic gland, no significant rescue was observed when *kdm5* was reintroduced into the corpus allatum. Similarly, expression of *kdm5* in the corpora cardiaca using Akh-Gal4 failed to rescue *kdm5^140^* mutants (Lee and Park, 2004) (Table 1). Thus, within the ring gland, KDM5 is crucial only for prothoracic gland function, linking its transcriptional regulatory functions to ecdysone biology.

**Fig. 3.**
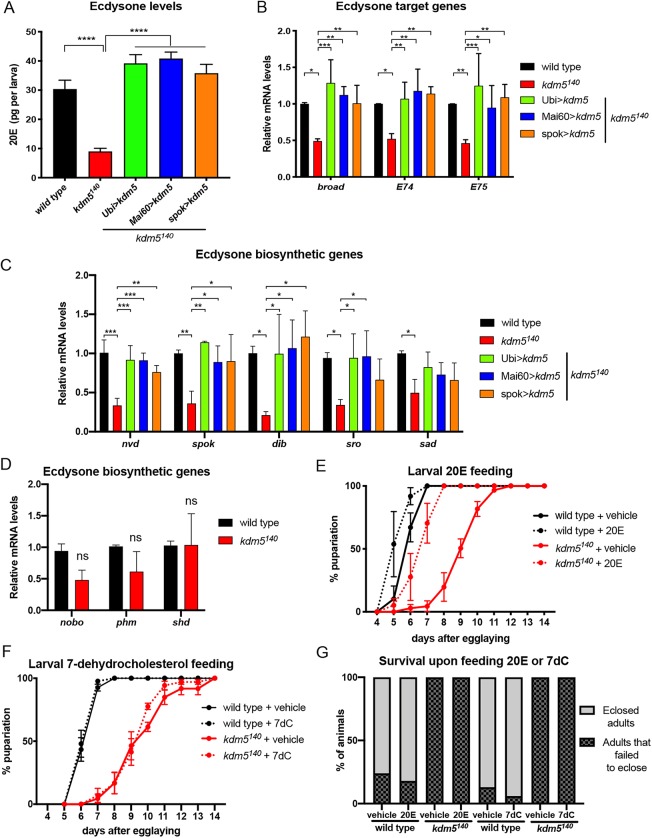
**Exogenous ecdysone rescues the developmental delay of *kdm5^140^* mutants.** (A) Quantification of 20E levels in pg per larva using wild-type, *kdm5^140^*, *kdm5^140^*; Ubi>*kdm5*, *kdm5^140^*; Mai60>*kdm5* and *kdm5^140^*; spok>*kdm5* whole 3rd instar larvae. ****P<0.001 (one-way ANOVA). Data are mean± s.e.m. (B) Real-time PCR of biological triplicate samples quantifying the mRNA levels of the ecdysone target genes *broad*, *E74* and *E75* in wild-type, *kdm5^140^*, *kdm5^140^*; Ubi>*kdm5*, *kdm5^140^*; Mai60>*kdm5* or *kdm5^140^*; spok>*kdm5* whole 3rd instar larvae. Data were normalized to *rp49* and are shown relative to wild type. *P<0.05, **P<0.01, ***P<0.001 (one-way ANOVA). Error bars indicate s.e.m. (C) Real-time PCR from biological triplicate samples quantifying the mRNA levels of the ecdysone biosynthetic genes *nvd*, *spok*, *dib*, *sro* and *sad* in wild-type, *kdm5^140^*; Ubi>*kdm5*, *kdm5^140^*; Mai60>*kdm5* or *kdm5^140^*; spok>*kdm5* whole 3rd instar larvae*.* Data were normalized to *rp49* and are shown relative to wild type. *P=0.05, **P<0.01, ***P<0.001 (one-way ANOVA). Error bars indicate s.e.m. (D) Real-time PCR of biological triplicate samples quantifying the mRNA levels of *phm*, *nobo* and *Shade* in wild-type or *kdm5^140^* whole 3rd instar larvae*.* Data were normalized to *rp49* and are shown relative to wild type. Error bars indicate s.e.m. No significant difference was observed (Student's *t*-test). (E) Quantification of the time for pupariation to occur upon feeding 20E to wild type (*n=*103) and *kdm5^140^* (*n=*62) or vehicle alone (ethanol) to wild-type (*n=*67) or *kdm5^140^* (*n=*60) animals. (F) Quantification of the time for pupariation to occur upon feeding 7-dehydrocholesterol (7dC) to wild type (*n=*214) and *kdm5^140^* (*n=*117) or vehicle alone (ethanol) to wild-type (*n=*198) and *kdm5^140^* (*n=*128) animals. Data are mean±s.e.m. in E,F. (G) Quantification of the number of pupae that eclosed from their pupal case for wild-type larvae that were fed vehicle alone (20E control, ethanol) (*n=*33) or 20E (*n=*40) or *kdm5^140^* larvae fed vehicle alone (20E control, ethanol) (*n=*16) or 20E (*n=*15), wild-type larvae that were fed vehicle alone (7dC control, ethanol) (*n=*73) or 7dC (*n=*68) or *kdm5^140^* larvae fed vehicle alone (7dC control, ethanol) (*n=*21) or 7dC (*n=*22). No statistical difference between vehicle and 20E or 7dC was observed for wild-type or *kdm5^140^* animals (Student's *t*-test).

Based on the requirement for KDM5 in the prothoracic gland, we tested whether *kdm5^140^* larvae had altered levels of the active form of ecdysone (20-hydroxyecdysone; 20E). These analyses revealed that *kdm5^140^* larvae that were matched to controls by developmental stage had a threefold reduction in 20E levels and concomitant reduction in the expression of ecdysone-regulated genes such as *broad*, *E74* and *E75* (Fig. 3A,B). Ecdysone is synthesized in the prothoracic gland from cholesterol through the action of a series of biosynthetic enzymes that include Noppera-bo (Nobo), Neverland (Nvd), Spookier (Spok), Shoud (Sro), Phantom (Phm), Disembodied (Dib) and Shadow (Sad) (Gilbert, 2004; Niwa and Niwa, 2014). qPCR analyses to examine the expression of the genes that encode these proteins revealed that *nvd*, *spok, sro*, *dib* and *sad* transcripts were significantly decreased in *kdm5^140^* mutant 3rd instar larvae (Fig. 3C). While *nobo* and *phm* trended toward being similarly reduced in *kdm5^140^* larvae, these did not meet statistical significance (Fig. 3D). We also examined levels of the hydroxylase Shade (Shd), which is necessary for converting ecdysone into 20E in peripheral cells of the larva, and found it to be unaffected by loss of KDM5 (Fig. 3D). *kdm5^140^* mutants are therefore likely to have reduced 20E owing to a defect in ecdysone biosynthesis in the prothoracic gland.

To test whether re-expression of *kdm5* in the prothoracic gland restored ecdysone production, we quantified levels of 20E in *kdm5^140^* larvae expressing UAS-*kdm5* using Ubi-Gal4, Mai60-Gal4 or spok-Gal4. All three Gal4 drivers restored levels of 20E, the expression of ecdysone-regulated genes and transcription of the ecdysone biosynthetic genes *nvd*, *spok*, *dib* and *sro* to wild-type levels (Fig. 3A-C). Based on these data, dietary supplementation with 20E but not the precursor molecule 7-dehydrocholesterol (7dC) would be expected to attenuate the phenotypes caused by loss of KDM5. Consistent with this prediction, exogenously providing 20E but not 7dC rescued the developmental delay of *kdm5* mutant larvae compared with vehicle-only controls (Fig. 3E,F). 20E was not, however, able to rescue the eclosion defect of *kdm5^140^* animals, whereas wild-type animals eclosed after ecdysone feeding (Fig. 3G). This could be because KDM5 is required for ecdysone production during pupal development when animals are unable to consume 20E-containing food. Alternatively, loss of KDM5 could cause additional defects in the prothoracic gland that are not able to be substituted by restoring 20E. As expected, based on its inability to rescue the developmental delay of *kdm5^140^*, 7dC supplementation did not alter the eclosion defect caused by loss of KDM5 (Fig. 3G).

### KDM5 regulates the Torso/MAPK signaling pathway and prothoracic gland endocycles

**Fig. 4.**
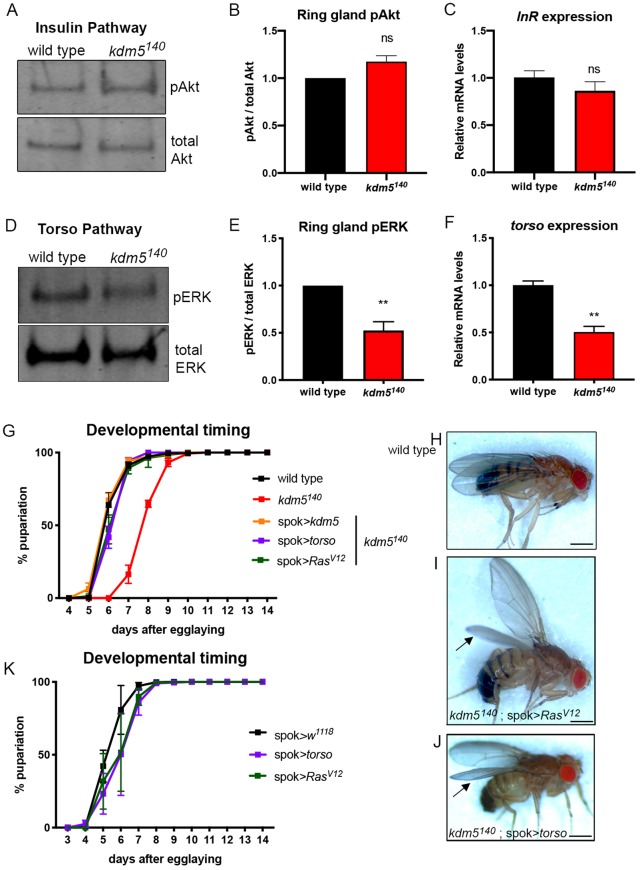
**Levels and activity of the Torso receptor pathway are reduced in *kdm5^140^*.** (A) Western blot analyses of wild-type and *kdm5^140^* dissected ring glands examining levels of phosphorylated Akt (pAkt; top) and total Akt (bottom) as a control. Six rings glands per lane. (B) Quantification of triplicate western blots showing the ratio of pAkt to total Akt in *kdm5^140^* compared with wild type. ns, not significant (Student's *t*-test). Error bars indicate s.e.m. (C) Real-time PCR of biological triplicate samples quantifying the average mRNA levels of *InR* in dissected brain-ring gland complexes from wild-type and *kdm5^140^* 3rd instar larvae. Data were normalized to *rp49* and shown relative to wild type. ns, not significant (Student's *t*-test). Error bars indicate s.e.m. (D) Western blot analyses of wild-type and *kdm5^140^* dissected ring glands examining levels of phosphorylated ERK (pERK; top) and total ERK (bottom) as a control. Five ring glands per lane. (E) Quantification of triplicate western blots showing the ratio of pERK to total ERK in *kdm5^140^* compared with wild type. **P<0.01 (Student's *t*-test). Error bars indicate s.e.m. (F) Real-time PCR from biological triplicate samples quantifying the average mRNA levels of *torso* in dissected brain-ring gland complexes from wild type and *kdm5^140^* 3rd instar larvae. Data were normalized to *rp49* and are shown relative to wild type. **P=0.0026 (Student's *t*-test). Error bars indicate s.e.m. (G) Number of days taken for pupariation to occur in wild-type (*n=*642) *kdm5^140^* (*n=*141), *kdm5^140^*; spok>*kdm5* (*n=*147), *kdm5^140^*; spok>*torso* (*n=*153) and *kdm5^140^*; spok>*Ras^V12^* (*n=*150) animals*.* (H) Male wild-type fly (*kdm5^140^*; g[*kdm5*:HA]attp86F). (I) Male *kdm5^140^*; spok>*torso* adult fly with normal body and curved down wings indicated by an arrow. (J) Male *kdm5^140^*; spok>*Ras^V12^* adult fly with normal body and curved wings indicated by an arrow. Number of days taken for pupariation to occur in wild-type control (spok>*w^1118^*; *n=*417), spok>*torso* (*n=*474) and spok>*Ras^V12^* (*n=*378) animals. Scale bars: 500 µm. Data are mean±s.e.m. in G,K.

To investigate the basis of the reduced ecdysone levels, we examined the activity of two key pathways known to regulate its production: the insulin receptor and Torso/MAPK pathways (Yamanaka et al., 2013). Activation of the insulin receptor by insulin-like peptides leads to the phosphorylation of Akt, which subsequently induces cell growth and ecdysone production (Danielsen et al., 2016; Ohhara et al., 2017). To test whether this pathway was affected by the loss of KDM5, we examined levels of phosphorylated Akt compared with total Akt in *kdm5^140^* ring glands by western blot (Fig. 4A,B). We also quantified mRNA levels of the insulin receptor (InR) in brain-ring gland complexes (Fig. 4C). Neither of these signaling components were affected in *kdm5^140^* larvae, suggesting that this pathway was unlikely to mediate the developmental delay caused by loss of KDM5.

Ecdysone production can also be regulated by activation of the Torso receptor by prothoracicotropic hormone (PTTH), which functions by activating a Ras/ERK MAP kinase cascade (Niwa and Niwa, 2014; Niwa et al., 2005; Yamanaka et al., 2013). To test whether the Torso/MAPK pathway was altered in *kdm5* null mutant larvae, we examined levels of phosphorylated ERK relative to total ERK in addition to mRNA levels of the *torso* receptor (Fig. 4D-F). Both phosphorylated ERK and *torso* expression were significantly decreased compared with wild type, suggesting that loss of KDM5 caused a defect in this pathway. To examine the functional role that the Torso/MAPK pathway played in the developmental delay and lethality of *kdm5^140^*, we expressed a UAS transgene encoding wild-type Torso or a constitutively activated form of the downstream GTPase Ras (Ras^V12^). Expression of *torso* or *Ras^V12^* using spok-Gal4 restored the developmental delay defect and also rescued the lethality of *kdm5^140^* to produce adults that showed a curved-down wing phenotype similar to that seen upon re-expression of *kdm5* (Fig. 4G-J; Table 3). Expression of *torso* or *Ras^V12^* using spok-Gal4 did not alter developmental timing in a wild-type background (Fig. 4K). These data are consistent with the reduced Torso/MAPK signaling observed in *kdm5^140^* playing a key role in mediating the prothoracic gland activities of KDM5.

**Table 3.**
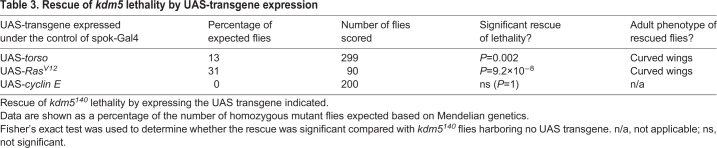
**Rescue of *kdm5* lethality by UAS-transgene expression**

**Fig. 5.**
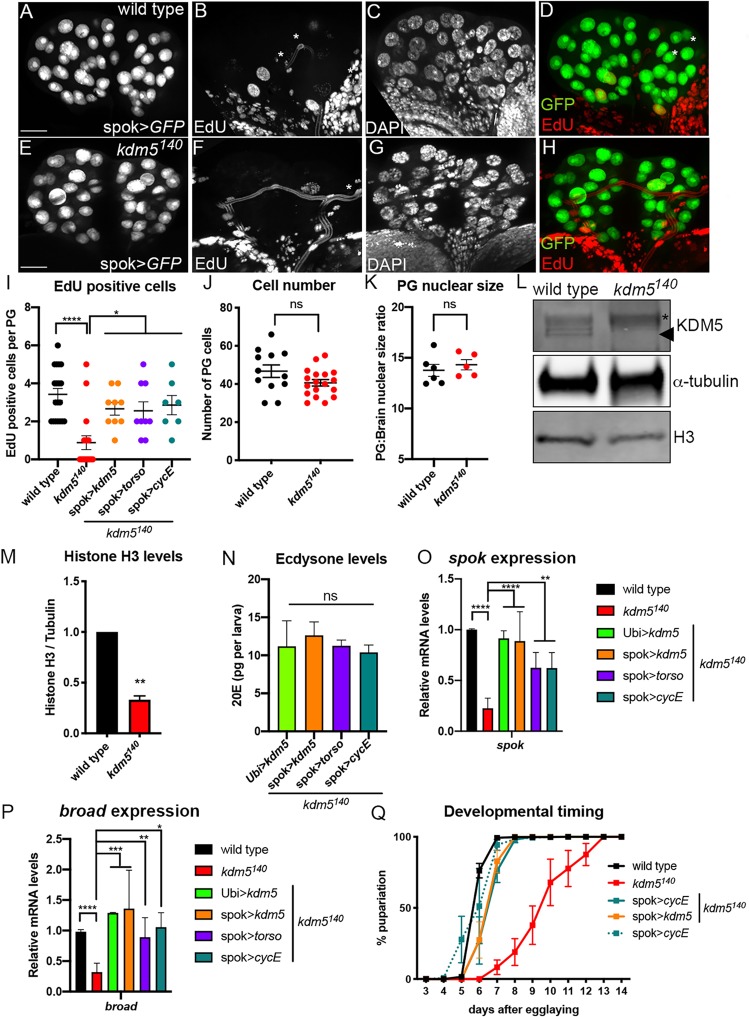
***kdm5^140^* larvae have a prothoracic gland endocycle defect that is rescued by expression of *cyclin E* or *torso*.** (A-D) Control larvae in which EdU incorporation was carried out using larvae from a cross of spok>GFP to the wild-type strain *w^1118^* to mark prothoracic gland cells. (A) GFP, (B) EdU, (C) DAPI and (D) merge of EdU and GFP channels. (E-H) *kdm5^140^* mutant larvae in which EdU incorporation was carried out using larvae of the genotype *kdm5^140^*; spok>GFP. (E) GFP, (F) EdU, (G) DAPI and (H) merge of EdU and GFP channels. Scale bars: 50 µm. (I) Quantification of the number of EdU-positive prothoracic gland nuclei from control larvae (*n=*7 larvae) and *kdm5^140^* homozygous mutant larvae (*n=*10), *kdm5^140^;* spok*>kdm5* (*n=*7), *kdm5^140^;* spok*>torso* (*n=*8) and *kdm5^140^;* spok*>cyclin E* (*n=*7). In all genotypes, prothoracic gland cells were marked using spok>GFP. ****P<0.0001, *P<0.05 (one-way ANOVA). Data are mean±s.e.m. with individual data points indicated (J) Quantification of the total prothoracic gland cell number in control spok>GFP (*n=*12 larvae) and *kdm5^140^*; spok>GFP (*n=*18) larvae. Data are mean±s.e.m. with individual data points indicated. ns, not significant (Student's *t*-test). (K) Quantification of prothoracic nuclear size expressed as a ratio to brain (diploid) nuclei from wild-type control spok>GFP (*n=*6) and *kdm5^140^*; spok>GFP (*n=*5) larvae. ns, not significant (Student's *t*-test). Data are mean±s.e.m. with individual data points indicated. (L) Western blot showing levels of KDM5 (top; indicated by arrowhead), α-tubulin (middle) and histone H3 from dissected ring glands from wild-type or *kdm5^140^* larvae. Asterisk indicates a non-specific band. (M) Quantification of three independent western blots comparing levels of histone H3 with α-tubulin. **P<0.01. Data are mean±s.e.m. (N) Quantification of 20E levels in pg per larva using *kdm5^140^*; Ubi>*kdm5*, *kdm5^140^*; spok>*kdm5*, *kdm5^140^*; spok>*torso* and *kdm5^140^*; spok>*cyclin E* whole 3rd instar larvae. Data are mean±s.e.m. No significant difference between the samples was observed (one-way ANOVA). (O) Real-time PCR from biological triplicate samples quantifying the mRNA levels of the ecdysone biosynthetic gene *spok* from wild-type, *kdm5^140^*, *kdm5^140^*; Ubi>*kdm5*, *kdm5^140^*; spok>*kdm5, kdm5^140^*; spok>*torso* and *kdm5^140^*; spok>*cyclin E* whole 3rd instar larvae. Data are normalized to *rp49* and are shown relative to wild type. **P<0.01, ****P<0.0001 (one-way ANOVA). Error bars indicate s.e.m. (P) Real-time PCR from biological triplicate samples quantifying the mRNA levels of the ecdysone target gene *broad* (*Br-C*) from wild-type, *kdm5^140^*, *kdm5^140^*; Ubi>*kdm5*, *kdm5^140^*; spok>*kdm5*, *kdm5^140^*; spok>*torso* and *kdm5^140^*; spok>*cyclin E* whole larvae. Data are normalized to *rp49* and are shown relative to wild type. *P,0.05, **P<0.01, ***P<0.001, ****P<0.0001 (one-way ANOVA). Error bars indicate s.e.m. (Q) Number of days taken for pupariation to occur in wild-type (*n=*261), *kdm5^140^* (*n=*56), *kdm5^140^*; spok>*kdm5* (*n=*73), *kdm5^140^*; spok>*cyclin E* (*n=*48) and spok>*cyclin E* (*n=*400) animals.

Torso/MAPK activation by PTTH has been linked to the regulation of prothoracic gland endocycling, which increases the DNA content of prothoracic gland cells up to ∼64C and is a key requirement for maximal expression of the genes that are limiting for the synthesis of ecdysone (Aggarwal and King, 1969; Ohhara et al., 2017; Rewitz et al., 2009; Shimell et al., 2018). Loss of KDM5 could therefore affect ecdysone production by affecting the ability of prothoracic gland cells to endoreplicate. To test this, we quantified the number of *kdm5* mutant prothoracic gland cells undergoing S phase by incorporating the thymine nucleoside analog 5-ethynyl-2’-deoxyuridine (EdU). This revealed a significant decrease in the number of EdU-positive cells in stage-matched *kdm5^140^* larvae compared with wild type (Fig. 5A-I). Importantly, loss of KDM5 caused these endocycle defects without altering the total number of cells that comprise the prothoracic gland (Fig. 5J). The specification of these cells therefore occurs normally during embryogenesis in *kdm5* mutants, but they fail to endocycle and grow correctly during larval development.

To further analyze the prothoracic gland endocycling defect of *kdm5^140^* larvae, we quantified nuclear size and levels of histone H3 in order to evaluate levels of polyploidization. These analyses revealed that the overall size of *kdm5^140^* prothoracic gland nuclei were similar to developmental stage-matched wild-type animals (Fig. 5K). However, western blot analyses of histone H3 from dissected ring glands from wild-type and *kdm5^140^* larvae revealed a significant decrease in the levels of this essential chromatin component (Fig. 5L,M). Because levels of histone H3 increase in-step with the increased amount of DNA generated with each endocycle, these data are consistent with *kdm5^140^* prothoracic gland cells having a polyploidization defect. Our observation that loss of KDM5 did not alter prothoracic gland nuclear size could additionally indicate a change in DNA compaction in these cells or that measuring nuclear size is not sensitive enough to detect modest changes to DNA content. Based on its ability to rescue the developmental delay of *kdm5^140^*, we next tested the extent to which *torso* expression in the prothoracic gland rescued the endocycling defect of *kdm5^140^*. Consistent with data linking the Torso/MAPK pathway to ecdysone production through promoting endoreduplication (Shimell et al., 2018), expression of *torso* using spok-Gal4 restored prothoracic gland endocycling in a similar manner to expression of *kdm5* (Fig. 5I). *torso* expression in *kdm5^140^* animals was also sufficient to rescue the expression of the ecdysone biosynthetic gene *spok*, levels of 20E and expression of the ecdysone response gene *broad* (Fig. 5N-P).

**Fig. 6.**
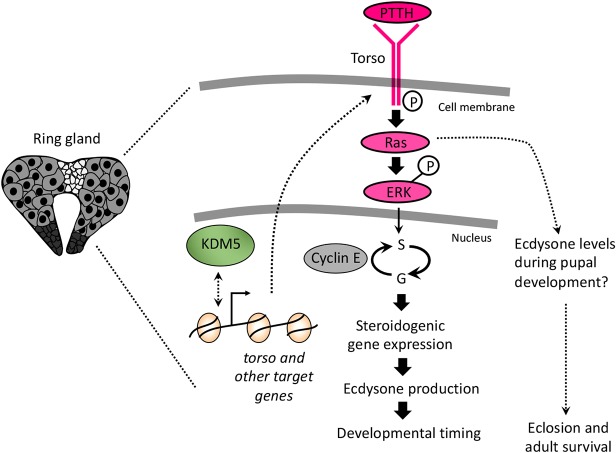
**Model for KDM5 function in the prothoracic gland.** We propose that KDM5 activates the Torso/MAPK pathway to regulate the expression of ecdysone biosynthetic genes and subsequent ecdysone production, in addition to other key cellular functions.

To directly explore the link between phenotypes caused by loss of KDM5 and reduced prothoracic gland endocycling, we drove endoreplication by overexpressing the cell cycle regulator Cyclin E (Ohhara et al., 2017). spok-Gal4-mediated expression of a UAS-*cyclin E* transgene in *kdm5^140^* mutant larvae rescued several key phenotypes, including their endoreplication defect, the slowed larval development, the levels of circulating 20E, and the expression of ecdysone biosynthetic and ecdysone inducible genes (Fig. 5I,N-Q). A deficit in prothoracic gland cell endocycling is therefore likely to be a key cause of the developmental delay seen in *kdm5^140^* larvae. Significantly, in contrast to activation of the Torso/MAPK pathway, expression of *cyclin E* was not able to restore viability to *kdm5^140^* mutant animals (Table 3). KDM5 is therefore likely to regulate larval growth rate by promoting ecdysone production through prothoracic cell polyploidization. In addition, KDM5 is needed for prothoracic gland function during larval and/or pupal development in a manner that is independent of this cell cycle activity (Fig. 6).

## DISCUSSION

In this study, we demonstrate that KDM5 is essential for the function of the ecdysone-producing prothoracic gland during *Drosophila* larval development. Crucial to this conclusion was our finding that expressing *kdm5* in the prothoracic gland was sufficient to rescue the lethality and developmental delay phenotypes of *kdm5^140^* null allele homozygous mutant animals. Consistent with this observation, prothoracic gland function was defective in *kdm5* mutants, with mutant larvae having low levels of ecdysone and reduced expression of downstream hormone-responsive target genes. Demonstrating the importance of KDM5-mediated regulation of ecdysone production, dietary supplementation of 20E restored normal developmental timing to *kdm5* mutant larvae. At the cellular level, loss of KDM5 slowed the prothoracic gland endoreplicative cycles that increase the ploidy of these cells and are important for ecdysone biosynthesis (Ohhara et al., 2017). Restoring these endocycles by expressing *cyclin E* re-established normal developmental timing to *kdm5^140^* mutants but was not able to rescue their lethality. In contrast, ectopic activation of the Torso/MAPK pathway that functions upstream of *cyclin E* was able to restore both developmental timing and rescue the lethality caused by loss of KDM5. We therefore propose that KDM5-mediated activation of the Torso/MAPK pathway in the prothoracic gland is important for larval growth regulation through its role in promoting polyploidization, and for adult eclosion by mechanisms that remain to be determined.

Consistent with an important developmental role for KDM5-mediated regulation of Torso/MAPK, mutations in *torso* or ablation of the neurons that produce its ligand PTTH cause a 5-day delay to larval development similar to that observed for *kdm5^140^* (Drelon et al., 2018; McBrayer et al., 2007; Rewitz et al., 2009). Because loss of KDM5 reduces, but does not eliminate, *torso* expression, the *kdm5^140^* phenotype cannot be accounted for solely by the twofold change to Torso/MAPK signaling observed. The dysregulation of additional KDM5-regulated genes is therefore likely to contribute to the prothoracic gland phenotypes of *kdm5* mutant larvae. Little is known about the transcriptional regulation of *torso* and other genes that make up the upstream pathways that regulate ecdysone production (Niwa and Niwa, 2016). One possibility is that KDM5 functions as a direct transcriptional activator of the *torso* gene; thus, decreased expression of this receptor would be expected in *kdm5* mutant animals. Because of the small size of the prothoracic gland, it is not currently feasible to carry out ChIP experiments to examine KDM5 promoter binding in this tissue. It is, however, notable that the *torso* promoter was not bound by KDM5 in existing ChIP-seq datasets from larval wing imaginal discs or from whole adult flies (Liu and Secombe, 2015; Lloret-Llinares et al., 2012). This could be because *torso* expression is largely restricted to the prothoracic gland during larval development and so may not be expected to have promoter-bound KDM5 in the tissues examined to date (Rewitz et al., 2009). Alternatively, KDM5 might regulate *torso* indirectly. In the silkworm *Bombyx mori*, expression of the *torso* gene is repressed in response to starvation conditions (Gu et al., 2011). Although the mechanism by which this occurs is unknown, it does indicate that other cellular defects caused by loss of KDM5 could lead to changes to *torso* transcription and subsequent decrease in MAPK activity and ecdysone production. Whether the regulation of *torso* by KDM5 is direct or indirect, it occurs in a demethylase-independent manner, as larvae lacking enzymatic activity show a normal developmental profile (Drelon et al., 2018). Consistent with this observation, components of KDM5 complexes that regulate gene expression through demethylase-independent mechanisms also affect developmental timing. For example, a development delay similar to that of *kdm5^140^* is caused by RNAi-mediated knockdown of the histone deacetylase *HDAC1* or the NuRD complex components *asf1* and *Mi-2* (Danielsen et al., 2016). KDM5 could therefore interact with these proteins to regulate the expression of genes that are crucial to the regulation of larval development.

Increased ploidy of prothoracic gland cells is important for optimal expression of steroidogenic genes and can be induced by activation of the Torso/MAPK pathway (Ohhara et al., 2017; Shimell et al., 2018). Because the genes required for ecdysone biosynthesis are among the most abundantly expressed in the prothoracic gland, their mRNA levels may be entirely limited by gene copy number (Christesen et al., 2017; Fox and Duronio, 2013). A similar requirement for copy number amplification to produce peak gene expression levels has been observed in other cell types in *Drosophila*, including chorion gene expression in ovarian follicle cells (Orr-Weaver, 2015). However, it is not known how Torso/MAPK activation promotes prothoracic gland cell cycle progression. One mechanism might be by affecting levels of cell cycle regulators such as the transcription factor E2f1, which is essential for both mitotic and endoreplicative cell cycles (Davoli and de Lange, 2011; Fox and Duronio, 2013; Frawley and Orr-Weaver, 2015). This model is based on studies of the polyploid enterocytes of the adult midgut, in which activation of the MAPK pathway via the EGF receptor stabilizes E2f1 protein, leading to transcription activation of *cyclin E* (Xiang et al., 2017). Although restoring correct endocycling in *kdm5* mutant prothoracic glands was able to rescue their developmental timing, it did not impact their eclosion defect. This could be because loss of KDM5 leads to additional defects within the prothoracic gland that are ultimately detrimental to the function of this endocrine tissue, such as increased oxidative stress, which we have shown to be affected in *kdm5* hypomorphic mutant wing discs (Liu et al., 2014). Alternatively, KDM5 could have a cell cycle independent role in maintaining ecdysone levels during pupal development. *kdm5^140^* mutant animals die as pharate adults that have no obvious morphological abnormalities but fail to eclose (Drelon et al., 2018). Nevertheless, these animals could have significant defects in, for example, nervous system development, which requires ecdysone and is important for eclosion (Boulanger and Dura, 2015; Syed et al., 2017).

Our observed role of KDM5 in the growth and polyploidization of larval prothoracic gland raises the possibility that it might play key roles in other cell types that use endoreplicative cycles. This could have broad consequences for our understanding of KDM5 biology, as polyploidization is observed in many plant and animal cell types and is widely used during *Drosophila* larval development (Fox and Duronio, 2013; Orr-Weaver, 2015). In addition, while the role of polyploid cells in the etiology or maintenance of cancers remains a topic of ongoing research, KDM5-regulated endocycling could contribute to its tumorigenic activities in humans. Regulation of polyploidization in cells of the nervous system could also contribute to the link between KDM5 protein dysregulation and intellectual disability (Vallianatos and Iwase, 2015). This could, for example, be mediated by KDM5 function in glial cells, as polyploidization of superineurial glial cells in *Drosophila* is required for normal brain development (Unhavaithaya and Orr-Weaver, 2012). Although it is not clear the extent to which a similar phenomenon occurs during human brain development, it is interesting to note that glial cell types contribute to the severity of intellectual disability disorders such as Rett syndrome (Sharma et al., 2018). Thus, although there is still much to be learned regarding the contribution of polyploid cells to normal development and to clinically relevant disorders, KDM5-regulated transcriptional programs could be key to the function of cells that use this variant cell cycle.

## MATERIALS AND METHODS

### Care of fly strains and crosses

Fly crosses were carried out at 25°C at 50% humidity and a 12 h light/dark cycle. Food (per liter) contained 18 g yeast, 22 g molasses, 80 g malt extract, 9 g agar, 65 cornmeal, 2.3 g methyl para-benzoic acid and 6.35 ml propionic acid. The sexes of dissected larvae for imaginal disc studies were not determined. For western blot and real-time PCR analyses, the number of male and female larvae were equal across the genotypes examined. For studies comparing wild-type and *kdm5* mutant larvae, we matched animals for developmental stage, and not chronological age, as we have done previously (Drelon et al., 2018). Thus, wild-type wandering 3rd instar larvae were ∼120 h after egg laying (AEL), while *kdm5^140^* larvae were ∼10 days old.

### Real-time PCR

Total RNA was purified from either whole wandering 3rd instar larvae or from dissected brain-ring gland complexes using TRIzol. Reverse transcription was carried out using 1 µg of RNA (or 5 µg of RNA for ecdysone biosynthetic genes) using a Verso cDNA kit (Thermo-Fisher AB1453A). Real-time PCR used the PowerUp SYBR Green Master Mix and was performed in Applied Biosystems Step ONE plus real-time PCR system. Changes to gene expression were determined by normalizing samples to *rp49* (*RpL32*).

### Western blot

Western blots were carried out with dissected wing discs or ring glands from developmental age matched 3rd instar larvae. Samples were dissected in 1×PBS and transferred to 1×NuPAGE LDS sample buffer, run on a 4-12% Bis-Tris 1 mm gel and transferred to PVDF. Secondary antibodies were donkey anti-mouse IgG or donkey anti-rabbit IgG 800CW. Blots were scanned using a LI-COR Odyssey Infrared scanner and quantified using LI-COR imaging software v3.0. Antibody sources and dilutions can be found in Table S1.

### Immunostaining of wing discs

3rd instar wing imaginal discs were dissected in 1×PBS and fixed in 4% paraformaldehyde for 30 min. After blocking in 0.1% BSA/TBST (1×PBS, 0.2% Triton and 0.1% BSA) for 1 h, samples were incubated with anti-Dcp-1 antibodies or anti-pH3 antibodies overnight at 4°C. Wing discs were washed with PBST and incubated with anti-rabbit Alexa Fluro 568 secondary antibody for 2 h at 4°C. Samples were mounted in Vectashield for microscopy. To compare wild-type and *kdm5^140^* staining, the number of Dcp-1- and pH3-positive cells within the pouch region of wing discs was quantified. We have previously shown that stage-matched *kdm5^140^* larvae have discs that are the same size as wild type (Drelon et al., 2018). Antibody sources and dilutions can be found in Table S1.

### Edu incorporation into larval tissues

For EdU staining, click-IT EdU kit and Alexa Fluor 594 Azide were used. Wild-type or *kdm5^140^* larvae that were matched for developmental time at the 3rd instar larvae were dissected in Schneider's *Drosophila* medium. Brain-ring gland complexes were then in incubated in 50 µM EdU in Schneider's Drosophila medium for 2.5 h at room temperature. After washing in 1×PBS, tissues were fixed in 4% paraformaldehyde for 30 min. Tissues were blocked in 3% BSA for 30 min and then permeabilized in 0.5% triton and detected using 1× click-IT reaction buffer, CuSO4, Alexa Fluor 594 Azide and reaction buffer additive for 30 min. DNA was stained using 4′,6-diamidino-2-phenylindole (DAPI) and samples were mounted in Vectashield for microscopy.

### Larval feeding of 20E or 7-dehydrocholesterol

Control wild-type or *kdm5^140^*/CyO-GFP flies were allowed to mate and lay eggs for 6 h. At 96 h after egg laying (AEL), a mixture of dry yeast, 20-hydroxyecdysone (0.33 mg/ml in 100% ethanol) or control ethanol alone in 500 µl of double-distilled H_2_O was added to each vial. For feeding 7-dehydrocholesterol (7dC), an established protocol was used in which a mixture of 50 mg yeast, 95 µl water and 5 µl 100% ethanol, and 0.75 mg 7-hydroxycholesterol was added to 30 h AEL larvae in vials that were shielded from light (Yoshiyama et al., 2006). *kdm5^140^* homozygotes were identified using the *Cy*O-GFP balancer chromosome.

### 20E quantification

Ecdysone was extracted from whole larvae as previously described (Moeller et al., 2017). Briefly, ten 3rd instar larvae were washed in double-distilled H_2_O and then homogenized in 0.5 ml methanol. After centrifugation, the pellet was re-extracted in 0.5 ml methanol and then in 0.5 ml ethanol. The three supernatants were mixed and 0.5 ml was evaporated using a SpeedVac. The pellet was dissolved in 100 µl of EIA buffer and subjected to the 20-hydroxyecdysone EIA Kit.

### Lifespan analyses

Lifespan analyses were carried out by collecting adult flies 24-36 h after eclosion and placing flies into vials at a density of 20 animals or fewer. Survival was quantified by counting the number of dead animals every day and transferring living flies into vials with fresh food every 2 days. Lifespan analyses were carried out using Log-rank (Mantel-Cox) test in Prism v8.0.

### Developmental delay quantification

Female and male flies were placed in a vial and allowed to lay eggs for 16 h. Starting at day 4 days AEL (or 3 days AEL for spok-Gal4 crosses in a wild-type background), the number of animals that had pupariated were scored every 24 h. Pupal genotype was ascertained by balancing *kdm5^140^* using a *Cy*O-GFP chromosome.

### Prothoracic gland size nuclear size determination

The ploidy of prothoracic gland nuclei was estimated by comparing their size with that of diploid brain nuclei, similar to published studies (Ohhara et al., 2017). Nuclear size of prothoracic gland and brain lobe nuclei were determined using Image J (Schneider et al., 2012). For each larva examined, the size of at least three prothoracic gland nuclei and three brain nuclei were averaged, and the ratio between the two tissues determined. At least five larvae were examined for wild type and *kdm5^140^*.

### Fertility analyses

To determine fertility, individual female flies were placed in a vial with five wild-type male flies and individual males were placed in a vial with three to five virgin wild-type females and allowed to mate and lay eggs for 4 days. If larvae were present in the vial by day 5, the fly was designated fertile. In cases where the fly died before day 3, we were unable confidently assess fertility and these animals were eliminated from the analyses.

### Image acquisition and processing

Adult fly images were obtained using Zeiss Discovery.V12 SteREO or ZEISS ApoTome microscopes and captured using AxioVision Release 4.8 software. Images of larval tissues were obtained using Zeiss AxioImager.M12 microscope and AxioVision SE64 Release 4.9.1 software. All images were processed using Adobe Photoshop CC 2019.

### Statistical analyses

All experiments were carried out in biological triplicate (minimum) and numbers (*n*) are provided for each experiment. Fisher's exact test was carried out in R. Student's *t*-test, one-way ANOVA and Wilcoxon rank-sum tests were carried out using GraphPad Prism.

### Resource and primer information

Resource and primer details can be found in Tables S1 and S2.
